# Refractive errors in a large dataset of French children: the ANJO study

**DOI:** 10.1038/s41598-022-08149-5

**Published:** 2022-03-08

**Authors:** Rébecca Guillon-Rolf, Leslie Grammatico-Guillon, Nicolas Leveziel, Francois Pelen, Eve Durbant, Jimmy Chammas, Raoul K. Khanna

**Affiliations:** 1 Department of Ophthalmology, Fondation Ophtalmologique Adolphe de Rothschild, Paris, France; 2 ANJO, Association Nationale Des Jeunes Ophtalmologistes, Centre Hospitalier National des 15-20, 28, Rue de Charenton, 75012 Paris, France; 3 Department of Medical Information, Centre Hospitalier Régional Universitaire, Bretonneau Hospital, Tours, France; 4 Department of Ophthalmology, Centre Hospitalier Universitaire, Poitiers, France; 5 Point Vision Centre, Paris, France; 6 Department of Ophthalmology, Centre Hospitalier Universitaire, Reims, France; 7 Neurogénétique et Physiopathologie Neuronale, iBrain, INSERM, U1253, Université de Tours, Tours, France; 8 Department of Ophthalmology, Centre Hospitalier Régional Universitaire, Bretonneau Hospital, Tours, France

**Keywords:** Paediatric research, Vision disorders

## Abstract

Undetected refractive errors (REs) in children can lead to irreversible vision loss. This study aimed to show the proportions of REs in French children using cycloplegic refraction. Multicentre cross-sectional retrospective study including children with cycloplegic refraction and without associated ocular conditions from 2015 to 2018 in French eye clinics. The following data were collected: age, symptoms of eye strain, best-corrected visual acuity (BCVA), cycloplegic refraction. The analysis included 48,163 children (mean age: 7.75 years, range: 2 to 12 years). The proportion of each RE was as follows: emmetropia (− 0.50 < Spherical Equivalent (SE) ≤  + 2.0; 58.3%), hyperopia (+ 2.0 $<$ SE $\le$+5; 17.2%), myopia (− 6 $\le$ SE $\le$− 0.50; 15.5%), high myopia (SE < − 6; 0.5%), high hyperopia (SE >  + 5; 3.6%), mixed astigmatism (4.9%). Anisometropia (SE difference ≥ 1.5) was found in 5.0%. Functional amblyopia in children attending primary school (aged over 6 years) was encountered in 2.7%. Symptoms of eye strain were frequent (70%) but not specific to any RE. REs are frequently found in French children and may remain undetected in the absence of symptoms of eye strain. Few studies have investigated REs in children using cycloplegic refraction, which has been shown to be the gold standard for RE assessment.

## Introduction

Undetected refractive errors (REs) remain a major cause of visual impairment worldwide^1^. Because of unequal access to care, screening for REs, prescriptions, and manufacture of corrective optical lenses may not be available in developing countries, and to a lesser extent, in developed countries. According to the World Health Organisation, 12 million children between 5 and 15 years of age have visual impairment caused by uncorrected REs^2^. Visual impairment is a major risk factor for amblyopia in these children, demonstrating the need to detect and manage REs during early childhood.

The prevalence of REs varies considerably worldwide. A review of 23 articles on REs in the Middle East concerning children under 15 years of age reported the following prevalence for each RE: myopia: 4% (spherical equivalent (SE) ≤  − 0.5 dioptres D); hyperopia: 8% (without cycloplegia, SE ≥  + 2.0 D), astigmatism: 15% (cylinder ≥ 0.75 D)^3^. In reality, this prevalence is inconsistent across countries owing to variable access to care, ethnic origin (i.e. higher prevalence of myopia in Asiatic children^4–6^), and environmental factors (i.e. higher prevalence of myopia in subjects with low outdoor/indoor activity ratios^7^ and in urban areas^8,9^). Age is also an important factor as it has an influence on the growth of the eye and the emmetropisation phenomenon. For instance, hyperopia decreases as age increases, with a prevalence of 5% at age 7, 2 to 3% between ages 9 and 14, and 1% at age 15^9^.

Studies vary worldwide on the use of cycloplegia to analyse the prevalence of REs. It is now well known that RE measurements are unreliable without cycloplegia, especially in children. Morgan et al*.* stated that cycloplegic refraction should be considered the gold standard for epidemiological studies^10^. Hashemi et al*.* highly recommends cycloplegic refraction for RE evaluation, especially in paediatric cases^10–12^.

This study aimed to show the proportions of REs in French children using cycloplegic refraction. The secondary objective was to evaluate the prevalence of amblyopia.

## Methods

### Study framework

This is a multicentre cross-sectional retrospective study including French children aged 2 to 12 years. Clinical data were collected from January 2015 to December 2018 from seven eye clinics (Versailles, Antony, Créteil, Poitiers, Troyes, Lyon Montrochet, Nice) considered as primary care centres and dedicated to REs. Appointments are mainly given online. First, patients are evaluated by an orthoptist who asks the parents for any general or ophthalmological personal history and then performs a best-corrected visual acuity (BCVA) assessment. Second, the ophthalmologist performs slit-lamp and fundus examination. All children are examined using cycloplegic drops as recommended by the French Association for Paediatric Ophthalmology and Strabismus.

The exclusion criteria were as follows: children over 12 years of age, which corresponds to the mean age of puberty in France^13^; consultations with a possibility of ocular disorder (e.g. retinal pathology, cataract, dyschromatopsia); missing autorefraction data; absence of cycloplegia; ophthalmic symptoms other than those of eye strain (e.g. red eye, painful eye, ophthalmic pruritus, ptosis).

This study was approved by the Ethics Committee of the French Society of Ophthalmology (Institutional Review Board: 0008855) and was conducted in accordance with the ethical principles of the Declaration of Helsinki. Data were anonymized for study purposes. Informed consent was not required for this study according to French law.

### Database composition and characteristics

The following data were extracted automatically from each patient data file (Ophtix© software, OPHTEL®): demographic characteristics including age at examination, any ophthalmological medical history, symptoms of eye strain (blurry vision, asthenopia, headache), the use of cycloplegic drops (cyclopentolate, Skiacol©, Alcon®, USA or Atropine®), autorefraction on both eyes (Tonoref©, Nidek®) after cycloplegia, and prescription of glasses at the end of the consultation. Patients were included if cycloplegic refraction was performed at the first visit, or within 6 months after the first visit in the case of delayed cycloplegic refraction.

The database contained anonymised administrative and ophthalmological data which were divided into quantitative (BCVA, cycloplegic refraction, refraction of prescribed glasses) and qualitative sections (ophthalmological medical history, reason for consultation, symptoms, slit-lamp and fundus examination). Quantitative variables were analysed directly, while for qualitative data, being textual, all words referring to ophthalmic disorders were excluded.

### Visual acuity measurement and refractive data

REs were classified according to the criteria described in Table 1, in accordance with a previous study^8^. Refractive status was defined according to SE. Anisometropia was defined as a difference in SE ≥ 1.5 D between both eyes^14^.

**Table 1:** Classification of refractive errors.

	Spherical equivalent (Dioptres)	Sphere (S, Dioptres)	Cylinder (C, Dioptres)
High hyperopia	SE > + 5	S $\ge$ 0	$\left|\mathrm{C}\right|\le \left|\mathrm{S}\right|$
Hyperopia^a^	+ 2.00 $<$ SE $\le$+5	S $\ge$ 0	$\left|\mathrm{C}\right|\le \left|\mathrm{S}\right|$
Emmetropia	− 0.50 < SE $\le$ +2.00		$\left|\mathrm{C}\right| \le 1.50$
Myopia^b^	− 6 $\le$ SE $\le$− 0.50	S $\le$ 0	
High myopia	< − 6	S $\le$ 0	
Mixed astigmatism		S $\ge$ 0	$\left|\mathrm{C}\right|>\left|\mathrm{S}\right|$ and $\left|\mathrm{C}\right|>$ 1.5

Absolute value * Negative cylinder notation was used to apply this classification.

^a^Including hyperopia and hyperopic astigmatisms.

^b^ Including myopia and myopic astigmatisms, *SE* spherical equivalent.

BCVA was assessed using decimal scales suitable to the child’s age using autorefraction or subjective refinement, where possible (Pigassou symbols in preschool children and Monoyer chart for schoolchildren). Decimal scale visual acuity was converted to logarithmic values using the following formula: log(1/BCVA). Amblyopia was defined as a difference in BCVA between both eyes for children above 6 years old as follows: mild (difference in logarithmic lines > 1 and < 3), moderate (difference in logarithmic lines ≥ 3 and < 10) and severe (difference in logarithmic lines ≥ 10)^15^.

Each child was first examined by an orthoptist who reported in each data file if there were any symptoms. If symptoms such as blurry vision (e.g. sensation of decreased visual acuity at distance or near fixation, difficulties in seeing the blackboard or reading) were reported, data was included in the “symptoms” section of each medical record.

### Statistical analyses

Statistical analyses were performed using the R software package version 3.6.0 (Rstudio®, Inc., Boston, MA, USA). Data were reported as the mean (SD) or median (interquartile range), as appropriate. For statistical independence purposes and based on the absence of significant differences between SEs for the two eyes, only right eyes were analysed, except for anisometropia for which both eyes had to be taken into account.

## Results

**Figure 1:**
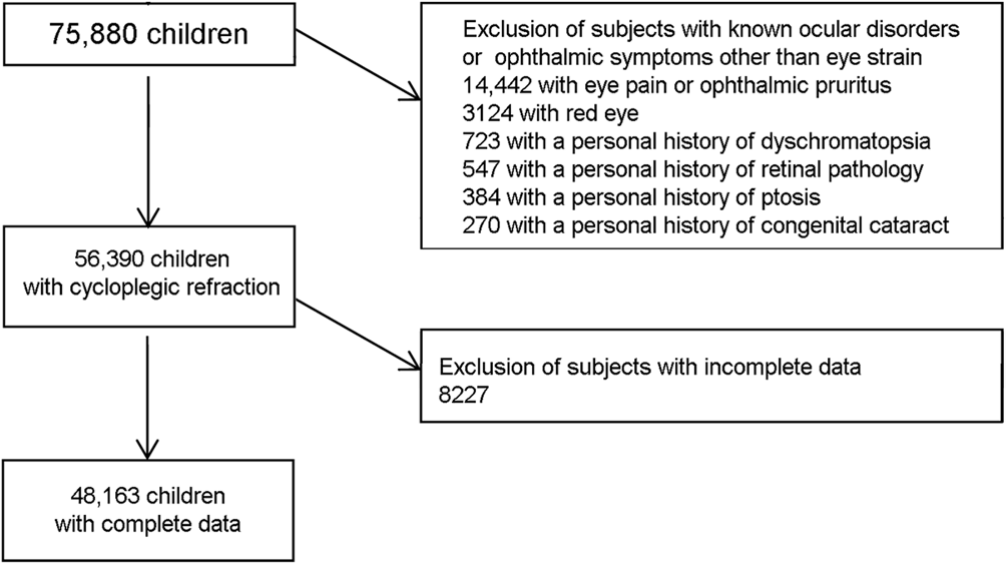
Study flowchart.

In total, 48,163 records met the inclusion criteria (mean age: 8 years, range: 2–12 years) (Fig. 1).

### Refraction

**Figure 2:**
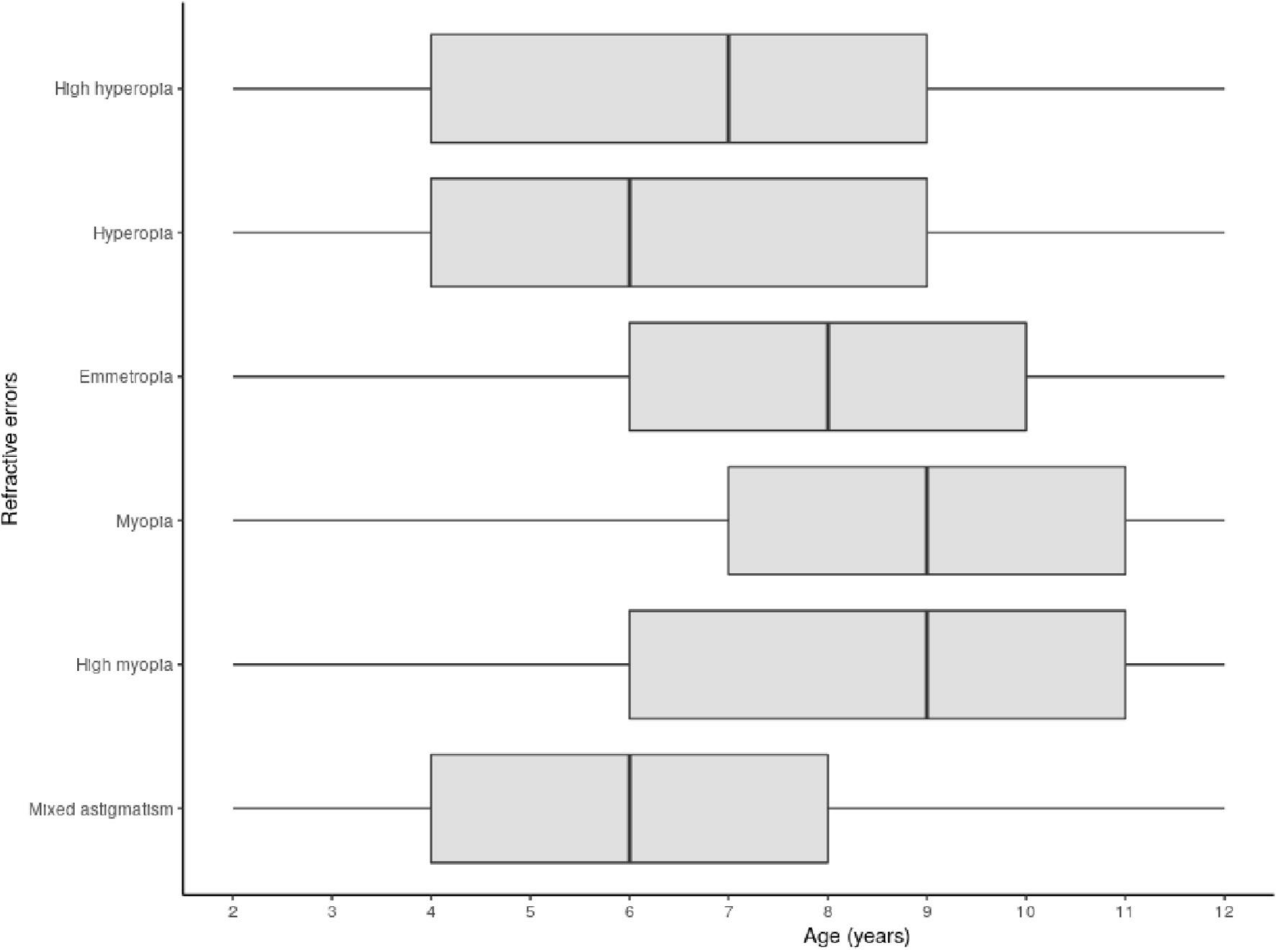
Boxplots of refractive errors according to age. Boxes indicate the interquartile range, central line: median value, whiskers: max and min values.

**Figure 3:**
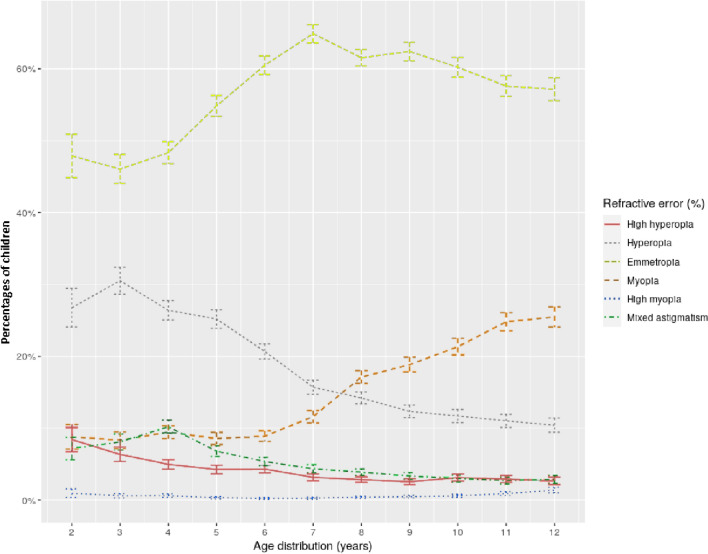
Evolution of the proportions of each refractive error according to age. Error bars represent standard deviations.

The proportions of each RE are indicated in Table 2. For participants aged 6 years old, the proportions of hyperopia and myopia were 20% and 10% respectively. These proportions increased to 12% and 19% in participants aged 9 years old. The number of children with hyperopia decreased with age, while those with myopia increased (Figs. 2 and 3). Hyperopia > 3.5 D was found in 2,273 children (4.7%). Anisometropia was found in 2,383 children (5.0%). In total, 22,537 (46.8%) children had a prescription for glasses at the end of the consultation.

**Table 2:** Number of subjects and values for each refractive error.

	Patients (N, % [95CI^c^])	SE^a^ (median, range, in D^b^)	Sphere (median, range, in D^b^)	Cylinder (median, range, in D^b^)
High hyperopia	1754 (3.6% [3.5; 3.8])	6.1 [5.1; 13.2]	7.0 [5.2; 13.5]	− 1. [− 5.8; 0]
Hyperopia	8275 (17.2% [16.8; 17.5])	2.9 [2.0; 5.0]	3.5 [2.0; 7.5]	− 0.8 [− 6; 0]
Emmetropia	28,066 (58.3% [57.8; 58.7])	0.0 [− 0.5; 1.9]	1 [− 0.2; 3.8]	− 0.2 [− 3.8; 0]
Myopia	7463 (15.5% [15.2; 15.8])	− 1.4 [− 6; − 0.5]	− 1 [− 5.8; 0]	− 0.5 [− 9.3; 0]
High myopia	266 (0.5% [0.5; 0.6])	− 7.8 [− 21.6; − 6.1]	− 7 [− 18.5; − 3.2]	− 1.5 [− 8.5; 0]
Mixed astigmatism	2339 (4.9% [4.7; 5.1])	0.1 [− 3.4; 2.9]	1.5 [0; 6.2]	− 2.8 [− 8.3; − 1.8]

^a^*SE* Spherical Equivalent.

^b^*D* Dioptres.

^c^*95CI* 95% confidence interval.

### Visual acuity and amblyopia

**Figure 4:**
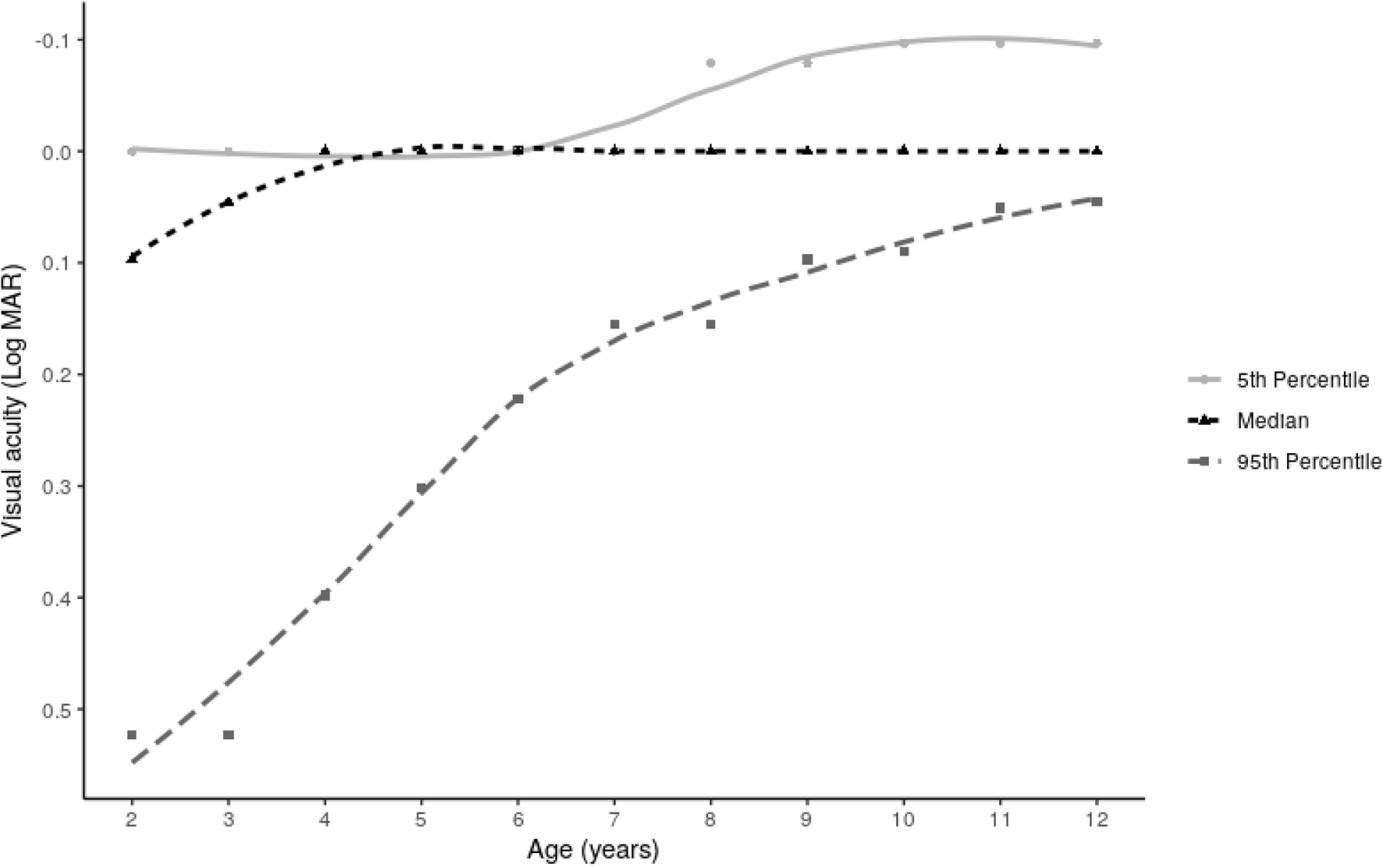
Best-corrected visual acuity according to age.

Age-specific BCVAs, with 5th, 50th, and 95th percentiles, are described in Fig. 4. From 2 to 4 years of age, the median BCVA increased to logMAR 0. At 6 years of age, 97.5% of the population had a BCVA higher than 20/32. At 9 years of age, 97.5% of the population had a BCVA higher than 20/25. In subjects over 6 years old, mild amblyopia was found in 815 (1.8%) children, moderate amblyopia in 447 (0.9%) children, while no severe amblyopia was reported.

### Symptoms of eye strain

Lastly, we analysed the visual symptoms according to the different REs (Table 3). A sensation of blurry vision was the most frequent symptom for all types of REs, from 62.3% for high hyperopia to 79.3% for myopia. Headache was the second most common symptom experienced by patients but much more rarely (ranging from 6.2 to 11.6%). Even in the emmetropia group, symptoms were not uncommon.

**Table 3:** Symptoms according to refractive error.

	Sensation of blurry vision	Asthenopia	Headache
High hyperopia (N, %)	1093; 62.3%	23; 1.3%	108; 6.2%
Hyperopia (N, %)	5464; 66%	193; 2.3%	764; 6.2%
Emmetropia (N, %)	20,633; 73.5%	1403; 4.9%	4765; 17%
Myopia (N, %)	5922; 79.3%	181; 2.4%	870; 11.6%
High myopia (N, %)	189; 71%	4; 1.5%	19; 7.1%
Mixed astigmatism (N, %)	1710; 73.1%	20; 0.9%	178; 7.6%

Percentages calculated on the number of patients in each refractive error category.

## Discussion

To our knowledge, this is the first study of a large dataset of French children using cycloplegic refraction. REs were found frequently, especially hyperopia and anisometropia, which are major risk factors for amblyopia.

It is now established that accommodation is an important interpretation bias when studying REs in paediatric populations. In studies without cycloplegic drops, accommodation can lead to overestimation of myopia and under-detection of hyperopia. Sankaridurg et al*.* highlighted the importance of considering cycloplegia to better describe each RE^11^. Hashemi et al*.* showed that cycloplegic refraction is more sensitive than subjective refraction in measuring REs^12^. Thus, the use of cycloplegic refraction in the current study is consistent with the recommendations of previous publications in order to reduce interpretation bias.

In this study, hyperopia (SE ≥ 2.0 D, including hyperopia and high hyperopia) was reported in 20.8% of children. The prevalence of hyperopia is highly heterogeneous within literature owing to the SE threshold value used for the definition. A review of 7 studies with systematic cycloplegia in Indian children from 0 to 15 years of age reported a prevalence of 4.7% for hyperopia (defined as SE > 2.0 D)^16^. In a literature review of 29 studies which included children over 5 years of age, the prevalence of hyperopia (> 2.0 D) ranged from 2.1% to 19.3%. In Denmark, significant hyperopia (≥ + 3.0 D) was found in 7.9% of children aged 4.5 to 7 years old^17^. When considering the criteria >  + 3.50 D for hyperopia at risk for amblyopia, proposed in the guidelines of the American Association for Pediatric Ophthalmology and Strabismus Vision Screening Committee, fewer children were identified (4.7%)^14^. The methodological characteristics of each study tend to make comparisons difficult. Moreover, it has been reported that populations with a Caucasian origin have the highest prevalence of hyperopia^18^.

Hyperopia can be asymptomatic when mild but can also be responsible for symptoms of eye strain, independently of its severity. It is therefore mandatory to consult an ophthalmologist if symptoms are present. In the current study, no symptom was specific to any RE. They are therefore all non-specific. Uncorrected hyperopia can cause amblyopia and impact vision-related quality of life or school performance^19^. Studies show that a well-corrected child performs better in school than an uncorrected child^9,20^. Hence systematic screening is necessary, and not only following the child’s complaints.

Myopia (SE ≤ − 0.50 D, including myopia and high myopia) was found in 16.0% of children, which is lower than those reported in Asian studies^4,21^. In a study performed in Shanghai, 52% of the children aged 10 had myopia^22^. Ethnic group can therefore have a significant impact. In the Netherlands, myopia prevalence was 11.5% at 9 years old^23^. Another study performed in 2015 in French children aged 9 years or younger reported a prevalence of 19.6% for myopia, which is consistent with our findings^24^. In our study, myopia was found in almost 1 out of 7 children, suggesting that it represents a public health issue. This is a major concern considering the potential increase of myopia in those children during puberty, especially with increasing time dedicated to indoor activities and a marked reduction of outdoor activities in developed countries^25^. Screening for myopia in children is very important in order to control environmental factors associated with myopia progression. There is currently an almost universal consensus that increasing outdoor activities is a protective factor. However, this does not occur with near-work activities, and although many researchers have confirmed the association with myopia, various epidemiological studies have not found such an association^26^.

Several studies have reported a prevalence of anisometropia ranging from 3.0% to 8.5% (in Australia, Afsari et al*.*: 3.0%^27^; in China, Hu et al*.*: 7.0%^28^; in Northern Ireland, O’Donoghue et al*.*: 8.5%^29^). Thus, the anisometropia prevalence of 5.0% in the current study is within the range of previous studies. The pathophysiological mechanisms underlying the development of anisometropia are still poorly understood. Yet, it is well known that anisometropia represents a major risk factor for amblyopia and impaired binocular vision and needs to be corrected^14^.

The prevalence of functional amblyopia in children attending primary school (6 years and older) was found to be 2.0%, which is consistent with previous findings (Faghigi et al*.*: 2.2% in children aged 5 to 15 years)^30^. In the absence of organic pathology, this represents a significant percentage of avoidable vision loss. Efforts have yet to be made to detect any RE at risk for amblyopia and we believe that systematic screening in pre-school children would be helpful.

Indeed, the representativeness of the subjects included is of great importance. To enable further comparisons with other countries, the characteristics of each healthcare system must be known. Specifically concerning the French vision healthcare system, children can be examined in private clinics, which represent primary care, or public hospitals, which represent secondary or tertiary care. Therefore, most children are examined by private ophthalmologists and then referred to public hospitals for further investigations for difficult cases. Second, visual screening is performed at the age of 3, which corresponds to the youngest children in the study population. Finally, these consultations are reimbursed by social security and mutual insurance and therefore the cost of care does not represent an obstacle to visual screening by an ophthalmologist. Any patient can make an appointment directly with an ophthalmologist for a visual examination in the current refractive centres, even without visual symptoms. Yet we cannot rule out that children with symptoms were more likely to be examined, as this study was retrospective. Further prospective studies with systematic screening would be relevant but it would hardly be possible to obtain such a large dataset. We also have to acknowledge that definitions of refractive errors are highly variable between studies, making comparisons difficult. Still, there is an increasing effort towards standardisation^31,32^, which remains an unmet need.

In conclusion, REs are frequently found in our cohort of French children, whether symptomatic or not. Symptoms of eye strain are non-specific, and the current findings highlight the need for systematic screening for REs in pre-school children in order to prevent irreversible vision loss and avoid low school performance. This is the largest public health study of REs using cycloplegic refraction in children.

## Data Availability

The datasets generated and/or analysed during this study are available from the corresponding author on reasonable request.
